# The effects of nitrogen form on root morphological and physiological adaptations of maize, white lupin and faba bean under phosphorus deficiency

**DOI:** 10.1093/aobpla/plw058

**Published:** 2016-08-12

**Authors:** Haitao Liu, Caixian Tang, Chunjian Li

**Affiliations:** 1 Department of Plant Nutrition, China Agricultural University, Beijing 100193, China; 2 Department of Animal, Plant and Soil Sciences, La Trobe University, Melbourne Campus, Bundoora, VIC 3086, Australia

**Keywords:** Low P availability, organic acids, rhizosphere, root exudation, root morphology, species variation

## Abstract

Root morphological/physiological modifications are important for phosphorus (P) acquisition of plants under P deficiency, but strategies differ among plant species. Detailed studies on the response of maize roots to P deficiency are limited. Nitrogen (N) form influences root morphology/physiology, and thus may influence root responses to P deficiency. This work investigated adaptive mechanisms of maize roots to low P by comparison with white lupin and faba bean supplied with two N forms. Plants were grown for 7–16 days in hydroponics with sufficient (250 µmol L^−1^) and deficient P supply (1 µmol L^−1^) under supply of NH_4_NO_3_ or Ca(NO_3_)_2_. Plant growth and P uptake were measured, and release of protons and organic acid anions, and acid phosphatase activity in the root were monitored. The results showed that P deficiency significantly decreased shoot growth while increased root growth and total root length of maize and faba bean, but not white lupin. It enhanced the release of protons and organic acid anions, and acid phosphatase activity, from the roots of both legumes but not maize. Compared with Ca(NO_3_)_2_, NH_4_NO_3_ dramatically increased proton release by roots but did not alter root morphology or physiology of the three species in response to low P. It is concluded that the N form did not fundamentally change root morphological/physiological responses of the three species to P deficiency. Morphological variation in maize and morpho-physiological modifications in white lupin and faba bean were the main adaptive strategies to P deficiency.

## Introduction

Low phosphorus (P) availability in the soil is one of the most limiting factors for crop production (Schachtman *et al.* 1998; Lynch 2007). Plants have evolved different mechanisms in roots in order to increase P acquisition under P-limiting conditions. These mechanisms include morphological modifications, mycorrhizal symbioses, rhizosphere acidification, release of carboxylates and phosphatases, and up-regulation of P transporters (Neumann and Römheld 1999; Raghothama 1999; Hinsinger 2001; Lambers *et al.* 2006). Root morphological variations are beneficial for labile P acquisition since P is relatively immobile in soil, and enhanced root growth increases exploration of localized P sources (Barber 1995); while root physiological modifications can increase mobilization of non-labile P in soil (Neumann and Römheld 1999; Hinsinger 2001).

Although the adaptive mechanisms of plants to P deficiency are well understood, relatively few studies have addressed the differences in root responses to P deficiency among plant species. White lupin (*Lupinus albus*), a model plant to study root response to P deficiency, shows a superior ability to utilize sparingly soluble P and bound P from soil by developing proteoid roots that exude protons (Dinkelaker *et al.* 1989), citrate and acid phosphatases (APase) (Dinkelaker *et al.* 1989; Gerke *et al.* 1994). Although faba bean (*Vicia faba*) does not form proteoid roots under P deficiency, its extensive root system, a morphological advantage, enables exploration of a larger volume of soil to access inorganic P (Nuruzzaman *et al.* 2005*a*, *b*). It also releases large amounts of protons, phenolics and APase which enhance mobilization of soil non-labile P and hence P uptake (Nuruzzaman *et al.* 2005*a*; Li *et al.* 2007).

Maize is widely cultivated, for both staple food and industrial usage, in tropical and temperate soils worldwide (Calderón-Vázquez *et al.* 2011). Results of the adaptive responses in maize roots to P deficiency, however, are controversial. Maize roots always showed an extensive morphological variation when the roots had restricted growth (Yan *et al.* 2011) and grew in nitrate-rich patches (Yu *et al.* 2013) and under P deficiency (Anghinoni and Barber 1980; Zhang *et al.* 2012). Phosphorus deficiency has been shown to either increase (Gaume *et al.* 2001) or decrease (Liu *et al.* 2004; Corrales *et al.* 2007) the release of organic acid anions from the roots in solution culture. It enhanced (Kummerová 1986) or did not change (Corrales *et al.* 2007) the activity of APase on root surface. Furthermore, P deficiency did not change rhizosphere pH (Neumann and Römheld 1999; George *et al.* 2002), or resulted in a strong alkalization in the rhizosphere (Li *et al.* 2007).

Nitrogen form influences the balance of cation and anion uptake by plant roots, and thus alters the rhizosphere pH. The change of rhizosphere pH caused by differential uptake of cations and anions is more prominent than that caused by root exudates such as protons and organic acid anions (Jaillard *et al.* 2003; Tang and Rengel 2003; Marschner 2012). Ammonium nutrition leads to preferential cation uptake and thus net proton excretion by roots, causing rhizosphere acidification; whilst nitrate supply induces hydroxyl secretion and causes rhizosphere alkalization (Gahoonia and Nielsen 1992; Mengel *et al.* 2001; Tang *et al.* 2011). In legumes, on one hand, N_2_ fixation generally leads to rhizosphere acidification due to excess uptake of cations over anions (Tang *et al.* 1997; Hinsinger *et al.* 2003; Li *et al.* 2008). On the other hand, N source is one of the factors affecting P supply of plants. Ammonium supply often enhances plant uptake of P from soil via rhizosphere acidification (Miller 1974; Marschne 2012). A lower pH in the rhizosphere could increase the H_2_${\text{PO}}_{4}^{-}$/${\text{HPO}}_{4}^{2-}$ ratio and the solubility of Ca-phosphates. However, interactions of N form and P supply on rhizosphere pH are not fully understood. For example, P deficiency could decrease, increase or have no effect on rhizosphere pH in ${\text{NO}}_{3}^{-}$-fed plants (e.g. Dinkelaker *et al.* 1989; Neumann and Römheld 1999; Tang *et al.* 2009). These different responses to P supply and N form depend on plant species.

The aim of this study was to elucidate the adaptive strategies of maize roots in response to P deficiency, and to investigate the influence of N form on these responses, using white lupin and faba bean as reference species.

## Methods

### Plant materials and treatments

Two hydroponic experiments were conducted in the greenhouse under natural light with 28 °C/16 °C day/night temperatures, and 45–55 % relative humidity. Each experiment consisted of three factors which were P supply (LP, 1 µmol L^−^^1^ P and HP, 250 µmol L^−^^1^ P), N forms [NH_4_NO_3_ and Ca(NO_3_)_2_], and plant species [maize (*Zea mays* L. cv. Zhengdan 958), white lupin (*Lupinus albus* L. cv. Amiga), and faba bean (*Vicia faba* L. cv. Lincan No. 5)]. There were four replicates for each treatment.

Seeds of the three plant species were surface-sterilized in 10 % (v/v) H_2_O_2_ for 30 min, rinsed thoroughly in distilled water and then germinated between filter papers moistened with saturated CaSO_4_ solution in the dark. After a week, seven seedlings of each species were transferred into 7-L opaque pots (21 cm in diameter and 24 cm in height) filled with nutrient solution of the following composition (mol L^−^^1^): K_2_SO_4_ 7.5 × 10^−^^4^, MgSO_4_ 6.5 × 10^−^^4^, KCl 1 × 10^−^^4^, H_3_BO_3_ 1.0 × 10^−^^6^, MnSO_4_ 1.0 × 10^−^^6^, CuSO_4_ 1.0 × 10^−^^7^, ZnSO_4_ 1.0 × 10^−^^6^, (NH_4_)_6_Mo_7_O_24_ 5.0 × 10^−^^9^ and Fe-EDTA 2.0 × 10^−^^4^ (Niu *et al.* 2007). Nitrogen was supplied as Ca(NO_3_)_2_ or NH_4_NO_3_ at the concentration of 2.0 × 10^−3^ mol L^−1^. Phosphorus was supplied as KH_2_PO_4_ at 1 or 250 µmol L^−1^, and KCl was added to the LP treatment to keep the same K concentration between the P treatments (Pang *et al.* 2010). The pH of nutrient solution was adjusted to 6.0 using 1 mol L^−1^ HCl or NaOH when the solution was renewed. The nutrient solution in each pot was continuously aerated and renewed every 3 days.

The first experiment was harvested 12 days after the commencement of treatments (DAT). Seven plants of each species in each pot were harvested as follows: (1) two plants were used to collect root exudates; (2) two plants were used to measure APase activity on root surface; (3) two plants were used to study rhizosphere acidification/alkalization using the agar technique; (4) one plant was used to measure rhizosphere pH using the scanning ion-selective electrode technique.

In the second experiment, nutrient solution pH was measured at 10:00 am daily during the experimental period using a pH meter (pH Testr30, USA). Plants were harvested 7, 12 and 16 days after the commencement of treatments. At each harvest, two roots of each species were kept at 4 °C for root length analysis. Five plants were separated into shoots and roots, and dried for dry weight and P measurements. In both experiments, no nodules were observed on roots of the two legumes, and no cluster roots were observed in white lupin at the last harvest (Wang *et al.* 2006).

### Collection of root exudates and analysis of organic acids

Plant roots were carefully rinsed three times with deionized water and were then immersed in 100 mL collection solution in an opaque vessel and aerated for 2 h from 10:00 am to 12:00 noon for collection of root exudates. Collection of root exudates under non-sterile condition reflects the root exudates in nature (Shen 1998). The compositions of the collection solution were (μmol L^−1^): 200 MgCl_2_, 100 KCl, 600 CaCl_2_ and 5 H_3_BO_3_. These nutrients in the collection solution allowed for membrane integrity and minimizing osmotic stress and possible passive leakage. After collection, a subsample of 8 mL of collection solution was acidified by adding two drops of concentrated H_3_PO_4_. Microbe inhibitor (Micropur, Katadyn, Deutsschl and Gmbh, München, Germany) was added into the collected exudate solutions at 0.01 g L^−1^ to prevent microbial decomposition of root exudates (Shen *et al.* 2004). The 8 mL of collection solution was then filtered through 0.2-μm membrane and frozen at −20 °C until analysis.

Organic acids were analyzed by a reversed-phase high performance liquid chromatography (Wang *et al.* 2006). Separation was conducted on a 250 × 4.6 mm reversed phase column (Alltima C-18). The mobile phase was 25 mmol L^−1^ KH_2_PO_4_ (pH 2.5) with the flow rate of 1 mL min^−1^ at 28 °C and UV detection was set at 214 nm. Organic acids in the sample were identified by comparison with the retention times and absorption spectra of pure standards including tartaric, malic, citric, fumaric and T-aconitic acids, which are the reported major organic acids released by white lupin, faba bean and maize (Dinkelaker *et al.* 1989; Neumann and Römheld 1999; Gaume *et al.* 2001; Liu *et al.* 2004; Li *et al.* 2007).

### Measurement of acid phosphatase activity

Plant roots were washed three times using deionized water, and blotted dry. The roots were placed in opaque vessels containing 0.5 g L^−^^1^ *p*-nitrophenyl phosphate (NPP) as a substrate in acetate buffer (0.1 mmol L^−^^1^, pH 5.6) for 1 h under nature illumination. Five mL of the reaction solution was mixed immediately with 2.5 mL NaOH (2 mol L^−1^) to terminate the reaction and to develop the colour (McLachlan 1980). The absorbance of the solution was determined at 405 nm using a spectrophotometer (UVmini 1240, Japan). The APase activity in the root was calculated on a basis of an oven-dried weight.

### Rhizosphere acidification/alkalization

Rhizosphere acidification/alkalization was detected using an agar technique modified from Marschner and Römheld (1983). Intact roots of the plants cultured in treatment solutions for 12 days were washed in deionized water (pH 5.9), spread out in a flat tray (310 mm × 150 mm) and covered immediately with the agar solution (0.7 %, 38 °C) containing pH indicator 0.01 % bromocresol-purple at pH 5.9. After 30 min, images were recorded. A yellow colour indicates acidification whereas purple colour means alkalization.

### Non-invasive measurement of H^+ ^fluxes on root surface

Net H^+ ^fluxes on root surface were measured using the scanning ion-selective electrode technique as described by Xu *et al.* (2006). Before measurement, microelectrodes were calibrated with pH 5.5 and pH 6.5 measuring solution, only those with a Nernst slope of 58 ± 6 mV were used. The H^+ ^flux was recorded every 6 s for 10 min. Data were treated using Mageflux software (Version 1.0) developed by Xuyue Company (http://xuyue.net/mageflux; Sun *et al.* 2009). The results were presented as pmol cm^−2^ s^−1^.

After washing in deionized water, the roots were placed in a beaker containing measuring solution (0.1 mmol L^−1^ KCl, 0.1 mmol L^−1^ CaCl_2_, 0.1 mmol L^−1^ MgCl_2_, 0.5 mmol L^−1^ NaCl, 0.3 mmol L^−1^ MES, 0.2 mmol L^−1^ NaSO_4_, pH 6.0) (Sun *et al.* 2009). After 20 min, the roots were transferred into a Petri dish containing fresh measuring solution. Net H^+ ^fluxes were continuously recorded for 10 min at 10 mm from the tip of lateral roots.

### Dry weight and P content measurement

The shoots and roots harvested from the second experiment were dried at 105 °C for 30 min and then at 70 °C to a constant weight. After dry weights were recorded, the materials were ground into powder. Subsamples of ground materials were digested with a mixture of concentrated sulfuric acid and 30 % H_2_O_2_ (v/v), and total P content determined using the molybdivanadate method (Soon and Kalra 1995).

### Root length analysis

The harvested roots were digitally scanned (Epson V700, Jakarta, Indonesia), and the resulting images were analyzed with the WinRHIZO version 5.0 (Regent Instruments, Quebec City, Canada). Axial root length was measured by a ruler before scanning, it stands for primary root length of legumes and the sum of axial root lengths of primary root, seminal roots and crown roots of maize. Lateral root density indicated the numbers of the first-order lateral roots per unit length of an axial root. Higher order lateral roots were not measured. Specific root length was the ratio of root length to root dry weight.

### Calculations

Root P influx between the two harvests (7 and 16 DAT) in the second experiments was calculated according to Brewster and Tinker (1972)

(1) $$In=\frac{\left(P_{2}-P_{1}\right)}{\left(t_{2}-t_{1}\right)}\times \frac{\;{\text{log}}_{e}\left(L_{2}/L_{1}\right)}{\left(L_{2}-L_{1}\right)}$$

where *In* refers to P influx (mg m^−1^ d^−1^), *P* refers to plant P content (mg plant^−1^), *t* refers to time (days) and *L* refers to root length (m plant^−1^). The indices *1* and *2* refer to 7 and 16 DAT, respectively.

Phosphorus-use efficiency (PUE) and P-absorption efficiency (PAE) were calculated according to Equations (2) and (3), respectively (Jakobsen *et al.* 1992; Corrales *et al.* 2007)

(2) PUE = total plant dry weight/total plant P content

(3) PAE = total plant P content/total root length

Root/shoot ratio was calculated according to Claassen and Jungk (1984)

(4) Root/shoot ratio=L/W

where *L* refers to root length (m plant^−1^) and *W* refers to shoot dry weight (g plant^−1^).

### Statistical analysis

The statistical means of different treatments were compared using the least significant difference (LSD) at a 0.05 level of probability using SAS 8.02. Differences between the P treatments were analyzed using the one-way PROC ANOVA. The effects of P supply and N forms on the variables and their interactions were tested using a two-way analysis of variance.

## Results

### Plant growth and P uptake

Low-P supply (1 µmol L^−1^) significantly decreased dry weights of maize at last two harvests, and of faba bean at the last harvest under both N forms, compared with HP (250 µmol L^−1^) supply (Table 1). These decreased plant dry weights were the consequence of the decreased shoot dry weights with the decrease being greater for maize than for faba bean **[see Supporting Information—Figure S1A and C]**. In contrast, LP increased root dry weights of the two species at three harvests under Ca(NO_3_)_2_, and of maize at the first two harvests under NH_4_NO_3_ **[see Supporting Information—Figure S1B and D]**. However, LP supply did not affect the dry weights of shoot or roots of white lupin at all three harvests irrespective of N form [Table 1; **see Supporting Information—Figure S1]**.

**Table 1.** Dry weights and P contents of the whole plant of white lupin, faba bean and maize grown with low (LP) (1 µmol L^−1^) and high P (HP) (250 µmol L^−1^) under two N forms [ca(NO_3_)_2_ and NH_4_NO_3_] for 7, 12 and 16 days.

N forms	P supply	White lupin			Faba bean			Maize		
		7 d	12 d	16 d	7 d	12 d	16 d	7 d	12 d	16 d
		Plant biomass (g dry weight per plant)								
Ca(NO_3_)_2_-N	LP	0.12	0.28	0.47	0.46	0.72	1.11	0.34	0.56	0.72
	HP	0.14	0.30	0.53	0.42	0.80	1.38	0.27	0.84	1.57
NH_4_NO_3_-N	LP	0.12	0.18	0.47	0.43	0.71	1.07	0.37	0.61	0.79
	HP	0.12	0.21	0.50	0.43	0.77	1.37	0.34	1.01	1.99
LSD (*P* = 0.05)		0.01	0.03	0.04	0.04	0.08	0.14	0.03	0.06	0.08
P level		n.s.	n.s.	n.s.	n.s.	n.s.	**	**	***	***
N form		n.s.	***	n.s.	n.s.	n.s.	n.s.	**	**	***
P × N		n.s.	n.s.	n.s.	n.s.	n.s.	n.s.	n.s.	*	***
		P content (mg P per plant)								
Ca(NO_3_)_2_-N	LP	1.04	1.62	1.67	3.39	4.06	4.33	0.89	0.73	0.75
	HP	1.48	2.34	3.96	5.37	8.34	15.46	4.38	10.29	19.90
NH_4_NO_3_-N	LP	1.05	1.18	1.67	3.53	4.60	5.01	0.97	0.87	0.96
	HP	1.34	1.88	4.69	5.98	8.62	15.79	5.47	15.06	25.11
LSD (*P* = 0.05)		0.12	0.26	0.38	0.60	1.52	2.03	0.62	1.28	2.17
P level		***	***	***	***	***	***	***	***	***
N form		n.s.	**	n.s.	n.s.	n.s.	n.s.	*	**	**
P × N		n.s.	n.s.	n.s.	n.s.	n.s.	n.s.	*	**	**

*P ≤0.05;

**P <* *0.01;

***P <* *0.001; n.s. not significant at *P *=* *0.05.

Nitrogen forms did not affect whole plant dry weights of white lupin and faba bean at three harvests with the exception at 12 days for white lupin, with significant lower dry weights under NH_4_NO_3_ than under Ca(NO_3_)_2_. In comparison, the plant dry weight of maize was significant higher under NH_4_NO_3_ than under Ca(NO_3_)_2_. The interaction between P levels and N forms on plant dry weight was only significant in maize at the last two harvests (Table 1).

Among the three species, total root length (TRL) was the greatest in maize and the smallest in white lupin. Compared with the HP treatment in maize, LP increased TRL, axial root length (ARL) and lateral root density (LRD) at all three harvests under Ca(NO_3_)_2_, and increased specific root length (SRL) at the last two harvests under Ca(NO_3_)_2_. Under NH_4_NO_3_ supply, however, LP increased TRL, ARL and LRD at first two harvests, while inhibited SRL of maize at the last harvest. For faba bean, LP increased TRL, due to the increased LRD at the last two harvests under Ca(NO_3_)_2_, while decreased TRL due to the decreased LRD at the last harvest under NH_4_NO_3_. In most cases, LP did not change the measured root length parameters of white lupin under both N forms compared with the HP treatment (Table 2).

**Table 2.** Root length parameters of white lupin, faba bean and maize grown with low (LP) (1 µmol L^−1^) and high P (HP) (250 µmol L^−1^) under two N forms [ca(NO_3_)_2_ and NH_4_NO_3_] for 7, 12 and 16 days.

Parameters	Ca(NO_3_)_2_ as N source						NH_4_NO_3_ as N source					
	White lupin		Faba bean		Maize		White lupin		Faba bean		Maize	
	LP	HP	LP	HP	LP	HP	LP	HP	LP	HP	LP	HP
7 days												
TRL (m)	1.6 b	2.0 a	4.6 b	6.1 a	14.5 a	12.4 b	1.8 a	1.7 a	4.7 a	4.8 a	20.9 a	14.9 b
ARL (cm)	28.3 b	31.5 a	32.3 a	36.0 a	251.6 a	219.5 b	23.8 b	26.9 a	26.1 a	29.7 a	221.4 a	189.9 b
LRD (no. cm^−1^)	2.6 a	2.4 a	1.4 b	1.7 a	4.0 a	3.4 b	2.7 a	2.1 b	3.5 a	2.5 b	3.9 a	3.4 b
SRL (m g^−1^)	41.2 a	41.4 a	27.0 a	33.5 a	111.3 a	113.6 a	43.7 a	38.0 b	28.5 a	28.0 a	159.0 a	147.9 a
12 days												
TRL (m plant^−1^)	3.9 a	4.9 a	11.0 a	8.8 b	33.6 a	24.5 b	3.9 a	4.3 a	9.0 a	9.8 a	47.4 a	26.9 b
ARL (cm)	36.0 a	35.7 a	38.7 a	42.4 a	497.4 a	336.8 b	34.9 a	38.9 a	39.0 a	41.2 a	420.5 a	301.7 b
LRD (no. cm^−1^)	3.9 a	2.9 b	2.0 a	1.6 b	3.7 a	3.5 b	3.2 a	3.5 a	2.8 a	2.9 a	4.8 a	4.1 b
SRL (m g^−1^)	86.0 a	73.9 b	36.7 a	43.0 a	176.2 a	150.0 b	82.4 a	80.7 a	42.8 a	36.8 b	226.0 a	216.1 a
16 days												
TRL (m)	6.2 a	6.3 a	23.5 a	13.1 b	43.2 a	36.2 b	5.8 a	6.9 a	10.7 b	18.2 a	52.7 a	50.5 a
ARL (cm)	52.9 a	48.6 a	51.8 a	1.6 b	506.8 a	454.3 b	36.6 b	46.8 a	46.8 a	46.2 a	508.3 a	450.1 a
LRD (no. cm^−1^)	3.7 a	3.5 a	1.8 a	1.6 b	4.0 a	3.5 b	3.6 a	3.5 a	2.1 b	2.8 a	4.5 a	4.4 a
SRL (m g^−^^1^)	74.6 a	68.1 a	73.6 a	63.8 b	196.9 a	189.1 b	79.7 a	82.5 a	56.2 b	72.7 a	201.1 b	269.7 a

Values of each pair in rows of individual plant species followed by different letters represent significant differences between the P treatments (*P *<* *0.05). Means ± SE, *n *=* *4. Abbreviations: TRL: total root length (m plant^−1^); ARL: axial root length (cm); LRD: lateral root density (number of lateral roots per unit axial root, no./cm); SRL, specific root length (m g^−1^).

All three species had significantly lower P content of the whole plant supplied with LP than with HP under both N forms. Similar to the influence on plant dry weight, N forms influenced plant P content only in maize, with more P uptake under NH_4_NO_3_, but not in two legumes. In maize, increasing P supply increased the P content more under NH_4_NO_3_ than under Ca(NO_3_)_2_, leading to an interaction between P levels and N forms (Table 1). Low P supply significantly decreased whole plant N and K contents of maize and faba bean at the last two harvests, but not of white lupin at either harvest, compared with the HP supply **[see Supporting Information—Table S1]**. Nitrogen forms significantly influenced whole plant N and K contents of the three species with few exceptions at the first harvest. NH_4_NO_3_ facilitated N uptake of all three species while Ca(NO_3_)_2_ facilitated K uptake of legumes. Unlike the legumes, maize took up more K under NH_4_NO_3_ than under Ca(NO_3_)_2_ at the last harvest, under both P levels **[see Supporting Information—Table S1]**.

### Plant P concentration and P efficiency

**Figure 1.**
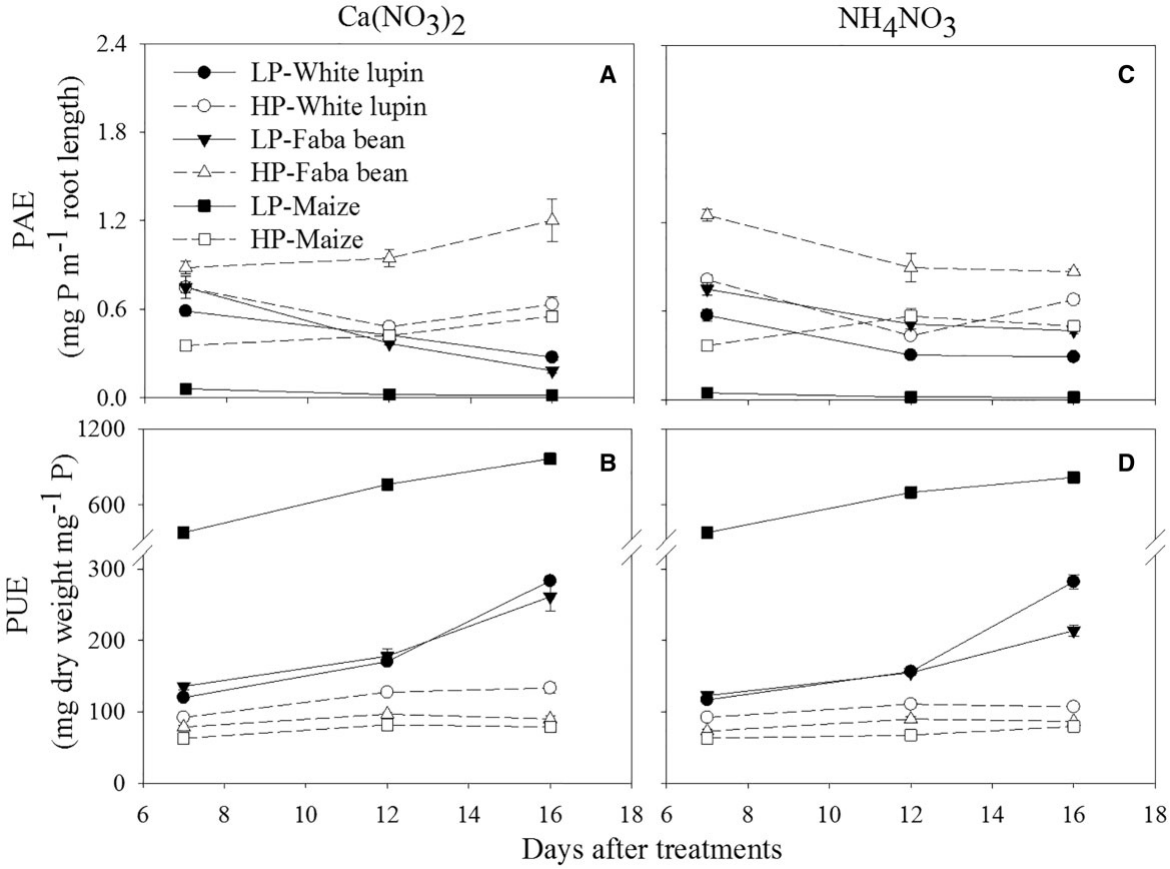
P-absorption efficiency (PAE) and P-use efficiency (PUE) of white lupin, faba bean and maize grown with low (LP) and high P (HP) under two N forms for 7, 12 and 16 days. Nitrogen was supplied as Ca(NO_3_)_2_ on left panels and NH_4_NO_3_ on right panels. Values are means of four replicates and error bars represent the standard error of the mean.

Compared with HP supply, LP significantly decreased plant P concentration (g P per 100 g DW) in all three species under both N forms. Maize showed the lowest P concentration under LP level while the highest value under HP level. Consistent with these results, maize had the lowest P-influx rate between the first and the third harvest (Table 3) and P-absorption efficiency (PAE) at three harvests under LP level and both N forms (Fig. 1A and C), when P influx and PAE were calculated per unit of root length. The P-influx rate was calculated by Equation (1), using the data in Tables 1 and 2. Because the P content of maize plants grown at LP did not increase between Days 7 and 16 (Table 1), the calculated P-influx rates of maize under LP and both N forms were zero (Table 3). However, irrespective of N form, maize had the highest P-use efficiency among the three plant species when LP was supplied (Fig. 1B and D). Nitrogen form did not affect P concentration in maize or faba bean at a given P level (Table 3).

**Table 3.** Plant P concentration and root/shoot ratio at the final harvest (16 days after treatment) and P influx rate between 7 and 16 days of white lupin, faba bean and maize grown with low (LP) and high P supply (HP) under two N forms [ca(NO_3_)_2_ and NH_4_NO_3_].

Parameters	Crops	Ca(NO_3_)_2_-N		NH_4_NO_3_-N		LSD	P level	N form	P × N
		LP	HP	LP	HP				
P concentration	White lupin	0.35	0.76	0.36	0.94	0.07	***	*	*
(g P 100 g^−1^ DW)	Faba bean	0.39	1.12	0.47	1.15	0.09	***	n.s.	n.s.
	Maize	0.10	1.27	0.12	1.26	0.05	***	n.s.	n.s.
P influx	White lupin	0.02	0.07	0.02	0.10	0.01	***	n.s.	*
(mg P m^−1^ root length d^−1^)	Faba bean	0.01	0.12	0.02	0.11	0.02	***	n.s.	n.s.
	Maize	0.00	0.08	0.00	0.08	0.01	***	n.s.	n.s.
root/shoot ratio	White lupin	16.5	15.6	15.6	18.1	1.8	n.s.	n.s.	n.s.
(m root length g^−1^ shoot DW)	Faba bean	31.3	12.2	14.9	18.0	3.5	***	**	***
	Maize	105.3	28.0	112.6	30.5	8.0	***	n.s.	n.s.

*P ≤0.05;

**P <* *0.01;

***P <* *0.001; n.s., not significant at *P *=* *0.05.

The supply of LP significantly increased the root/shoot ratio (m root length g^−1^ shoot DW) of maize under both N forms and of faba bean under Ca(NO_3_)_2_, but did not affect the root/shoot ratio of white lupin under either N form, compared with HP supply (Table 3).

### Proton release in roots of three plant species under two N forms

**Figure 2.**
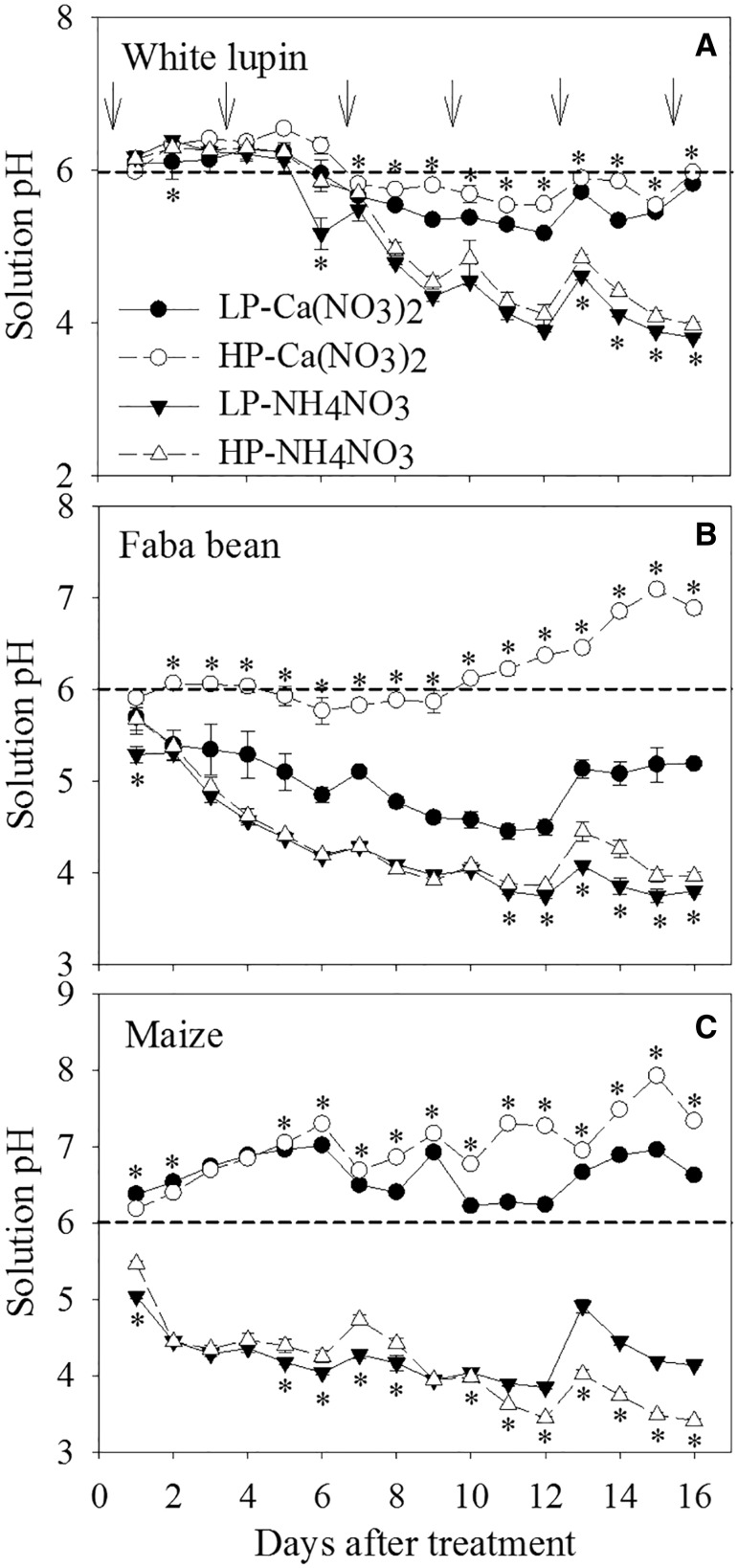
Changes over time in pH of nutrient solutions grown with white lupin, faba bean and maize supplied with low (LP) and high P (HP) under supply of Ca(NO_3_)_2_ or NH_4_NO_3_ during the cultural period. The pH was measured at 10:00 am daily. Arrows indicate the times when nutrient solutions were replaced. Values are means of four replicates, and the bars represent the standard error of the mean. Asterisks represent significant differences between the P levels at each measurement (*P *<* *0.05).

When Ca(NO_3_)_2_ was used as the N source, LP treatment increased proton release and decreased solution pH under both legumes, especially faba bean (Fig. 2A and B). In contrast, the solution pH under maize was higher than the initial pH value throughout the experimental period under both P levels, indicating an alkalization process (Fig. 2C). When NH_4_NO_3_ was supplied, the pH of solution grown with all three species declined dramatically over time with an exception of white lupin during the first 6 days (Fig. 2A). LP treatment further decreased solution pH under both legumes during the later cultural period, while increasing solution pH in maize during the same period. In comparison, the solution pH was lower with maize and faba bean than with white lupin under NH_4_NO_3_ supply (Fig. 2).

**Figure 3.**
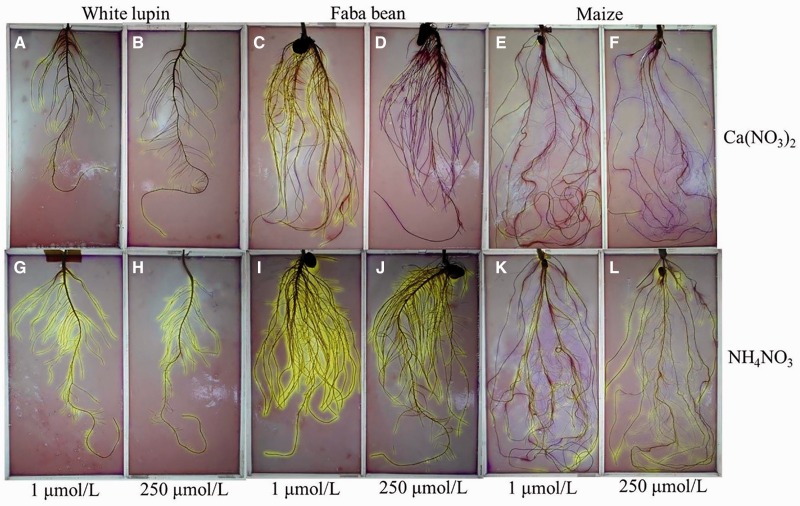
Effect of P supply (1 and 250 µmol L^−1^) on the intensity of rhizosphere acidification/alkalization in white lupin, faba bean and maize grown with Ca(NO_3_)_2_ or NH_4_NO_3_. Rhizosphere pH changes were detected by embedding the roots of 12-d-old plants in agar with bromocresol-purple as a pH indicator (initial pH 5.9). The images were recorded 0.5 h after embedding. Yellow colour indicates pH <5.2 while purple colour indicates pH >6.8.

**Figure 4.**
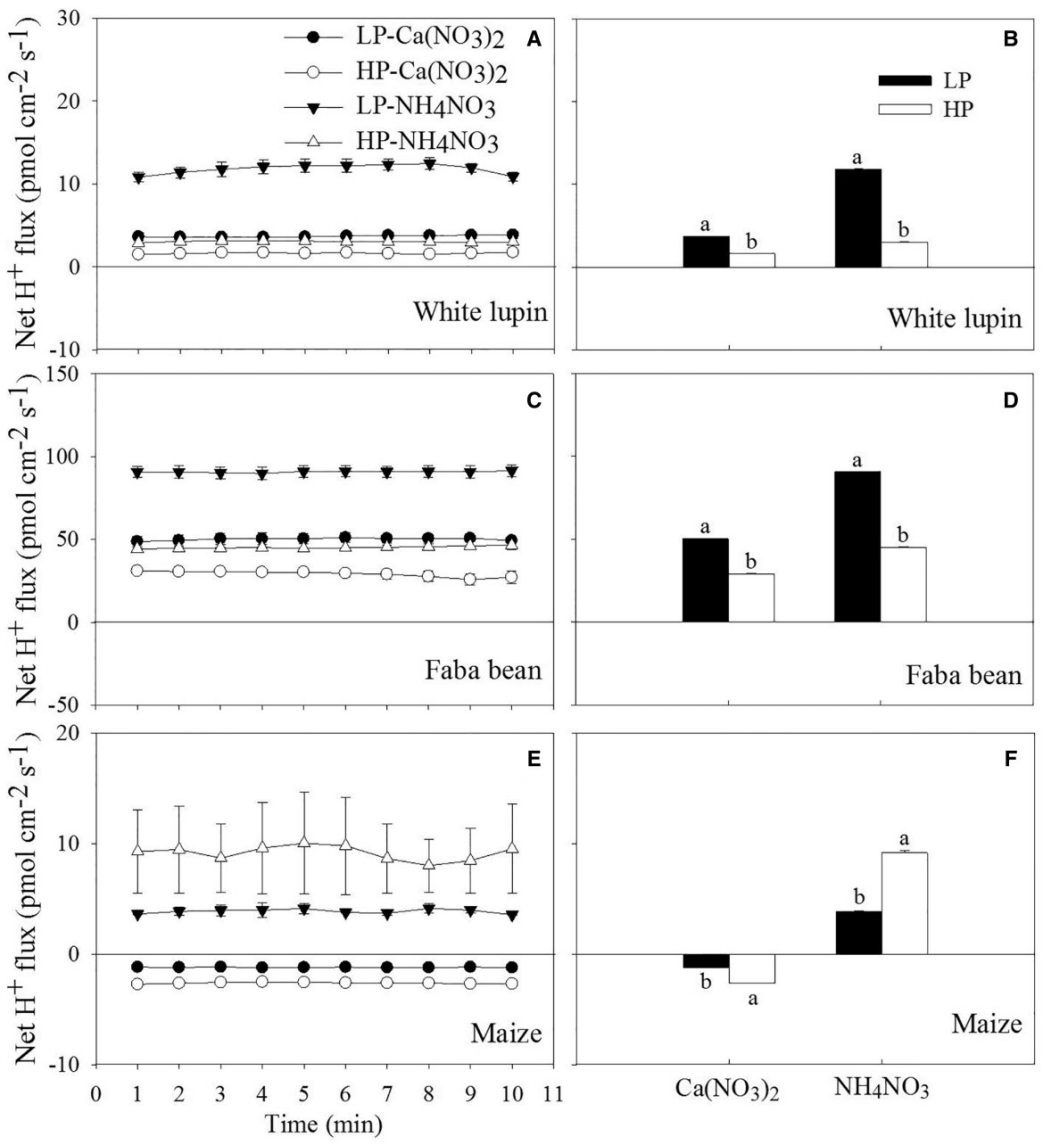
Net (left) and mean (right) H^+ ^fluxes over 10 min on root surface of white lupin (A and B), faba bean (C and D) and maize (E and F) that had grown for 12 days with low (LP) and high P (HP) supply under two N forms [Ca(NO_3_)_2_ and NH_4_NO_3_]. Values are means of four replicates, and the bars represent the standard error of the mean (A, C and E). Different letters (lower case) (B, D and F) represent significant differences between the P treatments within a plant species and N form (*P *<0.05). Positive values indicate net efflux while negative values indicate net influx.

Rhizosphere acidification/alkalization and net H^+ ^flux were compared among the three plant species on 12 days after treatments (Figs 3 and 4). When Ca(NO_3_)_2_ was supplied, yellow colour was observed surrounding the root tips of white lupin and LP-treated faba bean (Fig. 3A–C), indicating rhizosphere acidification, while purple colour along with the root surface of maize and HP-treated faba bean (Fig. 3D–F) revealed a rhizosphere alkalization. When NH_4_NO_3_ was supplied, yellow colour appeared on the root tips of maize, and on the surface of the entire root of the two legumes (Fig. 3G–L). The results were consistent with those of solution pH measurement (Fig. 2) and net H^+ ^flux determined using the scanning ion-selective electrode technique (Fig. 4). Proton release from the root tips was greater under NH_4_NO_3_ than under Ca(NO_3_)_2_ (Fig. 4).

### Root release of organic acid anions and acid phosphatase activity

Release of organic acid anions and APase activity on root surface were measured 12 days after treatments. The total amounts of organic acid anions released by the legume roots under both N forms were greater in the LP than in the HP treatment, where the increases of malate, citrate and tartrate in white lupin and of tartrate in faba bean were significant. In contrast, the release of organic acid anions by maize roots was lower in the LP than in the HP treatment (Table 4).

**Table 4.** Exudation of organic acid anions by roots of white lupin, faba bean and maize grown for 12 days with low (LP) and high P supply (HP) under two N forms [ca(NO_3_)_2_ and NH_4_NO_3_].

Crops	Organic acids	Root exudation (µmol g^−1^ dry root h^−1^)							
		Ca(NO_3_)_2_-N		NH_4_NO_3_-N		LSD	P level	N form	P × N
		LP	HP	LP	HP				
White lupin	Tartaric	0.96	0.54	0.49	0.50	0.04	***	***	***
	Malic	1.67	0.92	1.66	1.24	0.27	**	n.s.	n.s.
	Citric	0.59	0.47	n.d.	n.d.	0.04	***	*	*
	Fumaric	0.02	0.03	n.d.	n.d.	0.00	n.s.	n.s.	n.s.
	T-aconitic	0.04	n.d.	n.d.	0.03	0.00	n.s.	n.s.	***
	Total organic acids	3.29	1.95	2.15	1.82	0.29	***	**	**
Faba bean	Tartaric	1.35	0.83	2.87	2.25	0.20	***	***	n.s.
	Malic	0.75	0.81	0.89	0.79	0.39	n.s.	n.s.	n.s.
	Citric	0.31	0.42	0.48	0.35	0.15	n.s.	n.s.	n.s.
	Fumaric	0.05	n.d.	n.d.	n.d.	0.02	**	**	**
	T-aconitic	n.d.	0.01	n.d.	0.02	0.00	***	**	**
	Total organic acids	2.47	2.08	4.24	3.81	0.35	**	***	n.s.
Maize	Tartaric	0.41	3.62	0.49	2.19	1.08	**	n.s.	n.s.
	Malic	n.d.	1.20	n.d.	0.91	0.27	***	n.s.	n.s.
	Citric	n.d.	n.d.	n.d.	n.d.	0.00	n.s.	n.s.	n.s.
	Fumaric	0.03	0.05	0.02	0.02	0.01	n.s.	*	n.s.
	T-aconitic	0.48	0.29	n.d.	n.d.	0.15	n.s.	***	n.s.
	Total organic acids	0.92	5.16	0.51	3.12	1.42	**	n.s.	n.s.

*P ≤0.05;

**P <* *0.01;

***P <* *0.001; n.s., not significant at *P *=* *0.05. n.d., not detected.

**Figure 5.**
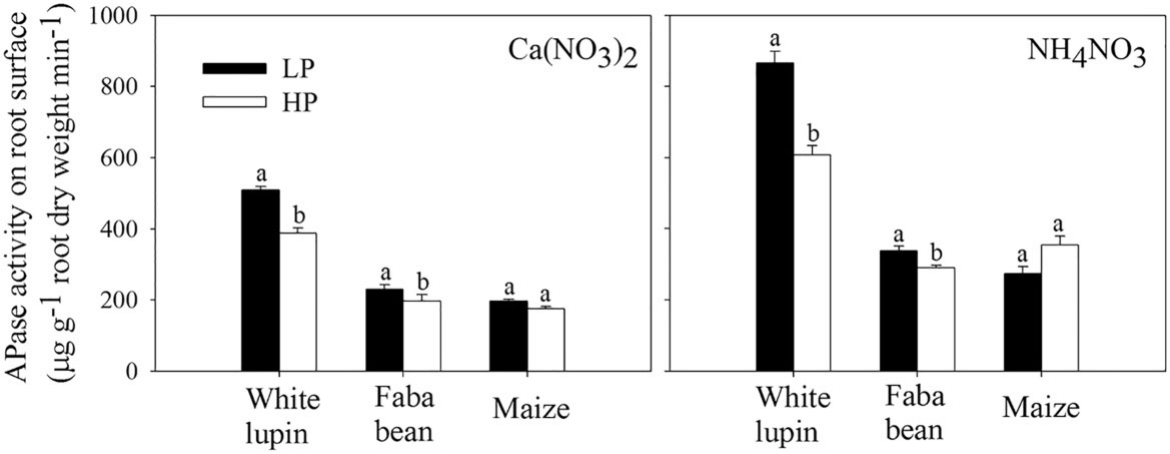
Activity of acid phosphatase (APase) on the root surface of white lupin, faba bean and maize grown for 12 days with low (LP) and high P (HP) supply under two N forms [Ca(NO_3_)_2_ and NH_4_NO_3_]. Values are means of four replicates and the error bars represent the standard error of the mean. Different letters (lower case) represent significant differences between the P treatments within a plant species and N form (*P *<* *0.05).

Low P supply significantly increased the APase activity on root surface of the legumes but not maize. The APase activity on root surface was higher under NH_4_NO_3_ than under Ca(NO_3_)_2_ for all three species, and was higher in white lupin than in faba bean and maize (Fig. 5).

## Discussion

### Morphological modification was the main adaptive response of maize roots to P deficiency

This study demonstrated that the growth of maize was more sensitive to P deficiency than that of the two legumes. This is consistent with the findings that maize has a high soil-fertility requirement to attain maximal grain yield (Paponov and Engels 2003). Under P deficiency, more carbon was distributed to roots and was used for the formation of new roots, but not for increases in the release of protons, organic acid anions and APase activity although the two N forms had contrasting effects on net H^+ ^release (Figs 2–5 and Table 4). These responses were also true for different maize genotypes (Anghinoni and Barber 1980; Corrales *et al.* 2007; Li *et al.* 2012; Fernández and Rubio 2015) under a broad range of growing conditions, including different P levels and growth media like hydroponics (Anghinoni and Barber 1980; Mollier and Pellerin 1999; Gaume *et al.* 2001; Li *et al.* 2012; Fernández and Rubio 2015), sand culture (Corrales *et al.* 2007) and field conditions (Fernández *et al.* 2009; Zhang *et al.* 2012). Additionally, the density and the length of maize root hairs were also found to increase under P deficiency (Zhu *et al.* 2005). While distributing more carbon to roots increases root length and surface area for exploration and acquisition of P from the soil (Tang *et al.* 2009), P-deficiency decreased photosynthesis and reproductive growth of the plant (Lynch and Ho 2005). In fact, low P supply dramatically decreased maize shoot growth [Table 1; see **Supporting Information—Figure S1**]. In other studies, continuous P deficiency decreased the growth of maize shoot and roots, and subsequently grain yields (Plénet *et al.* 2000; Zhang *et al.* 2012; Krey *et al.* 2013; Deng *et al.* 2014). It is not clear why maize has the priority to invest more carbon for root morphological over physiological modifications under P deficiency.

Maize responded to P deficiency similarly to wheat and does not increase proton release or exudation of organic acid anions in roots (Neumann and Römheld 1999; Li *et al.* 2008). In contrast, maize and wheat roots exhibit a remarkable increase in total root length and root/shoot ratio in response to P deficiency (Table 3; Vlek *et al.* 1996; Bhadoria *et al.* 2002). A larger root system is obviously beneficial for a better access of inorganic P because of the low mobility of P in the soil (Hinsinger 2001). Phosphorus uptake by plants depends on the root length and surface area, and on lateral roots to explore a large soil volume (Richardson *et al.* 2009; Balemi and Negisho 2012; Lambers *et al.* 2015). According to the results in the literature and the present study, it is concluded that the main adaptive strategy of maize roots to P deficiency is morphological variation.

### White lupin and faba bean exhibited morpho-physiological strategy in roots in response to P deficiency

Unlike maize, low P did not influence shoot and root growth of white lupin under both N forms in the present study **[see Supporting Information—Figure S1]**, although the shoot and root P content and concentration decreased (Tables 1 and 3). In comparison, low P increased the root dry weight, total root length and root/shoot ratio of faba bean under Ca(NO_3_)_2_ supply **[****Tables 2 and 3****; see Supporting Information—Figure S1]**, indicating that faba bean has the morphological advantage of an extensive root system favouring to access P by exploring a larger volume of soil. The P concentration of white lupin and faba bean was higher than that of maize under low P supply (Table 3), indicating that both legumes have a higher internal P requirement than maize (Föhse *et al.* 1988).

It is well known that P-deficient white lupin adapts P deficiency by morphological and physiological variations, i.e. formation of cluster roots and increased release of organic acid anions and protons (Dinkelaker *et al.* 1989; Gerke *et al.* 1994). In the present study, white lupin supplied with low P had not yet formed cluster roots because the duration of low-P treatment was not long enough (Dinkelaker *et al.* 1989; Gerke *et al.* 1994; Neumann and Martinoia 2002; Wang *et al.* 2006). Furthermore, malate was the main organic acid anion released by the roots (Table 4), yet the major organic acid anion released by root clusters is citrate while that released by root tips, including non-proteoid roots and proteoid roots, is malate (Gardner *et al.* 1982, 1983; Neumann *et al.* 1999). Apparently, white lupin roots adapted P deficiency by morphological/physiological modifications, although the carbon costs for root physiological variation are greater than that for morphological modification (Gardner *et al.* 1983; Dinkelaker *et al.* 1989; Lynch and Ho 2005). Since white lupin grows in P-impoverished habitats, the best strategy of the P-deficient white lupin is to mine soil P by releasing protons and organic compounds, rather than modifying root growth (Neumann and Martinoia 2002; Lambers *et al.* 2008, 2011).

Although reflecting different aspects of proton release by roots, the results obtained using solution pH measurement, agar technique and non-invasive measurement of net H^+ ^fluxes were consistent and showed that low P stimulated proton release in the two legumes, in agreement with the previous findings (Dinkelaker *et al.* 1989; Nuruzzaman *et al.* 2005*a*). The proton release induced by P deficiency was greater in faba bean than in white lupin, as presented by the intensive rhizosphere acidification within 30 min (Fig. 3) and more H^+ ^efflux on root tips of faba bean (Fig. 4). On one hand, the increased proton release by the roots of P-deficient faba bean would contribute greatly to mobilization of non-labile P in soil, especially in high-pH soils (Li *et al.* 2004). On the other hand, the increased release of protons under P deficiency may compensate for both excess uptake of cations (Dinkelaker *et al.* 1989) and a concomitant release of organic acid anions (Neumann and Römheld 1999).

### Nitrogen form did not affect the root morphological/physiological response to low P

The present study investigated whether N forms influence root responses to P deficiency. NH_4_NO_3_ facilitated N uptake of all three species while Ca(NO_3_)_2_ facilitated K uptake of legumes **[see Supporting Information—Table S1]**. In comparison with Ca(NO_3_)_2_, NH_4_NO_3_ supply resulted in an increase in proton release from the roots of all three species, especially maize under the high-P condition. Low-P supply increased proton release in white lupin and faba bean at the supply of both N forms, and in maize at the supply of NH_4_NO_3_ (Figs 2–4). The marked increase in proton release of the NH_4_NO_3_-fed maize under both P levels could be explained by the preferential uptake of ${\text{NH}}_{4}^{+\;}$over ${\text{NO}}_{3}^{-}$ under mixed supply of ammonium and nitrate, which causes more proton release (Clark 1982). There is a competition for transport across the tonoplast between chloride and nitrate, which affects their accumulation and uptake (White and Veneklaas 2012). In the present study, KCl was added to the low-P treatment to keep the K supply constant between the P treatments, which increased Cl concentration (250 µmol) in the low-P solution (see Methods section). In comparison with the high-P treatment, however, low P did not decrease N uptake in white lupin, but decreased it in faba bean at the final harvest and maize at the last two harvests under both N forms **[see Supporting Information—Table S1]**. The increased uptake of N could be explained by the larger plant biomass and thus high demand of the faba bean and maize supplied with high P (Table 1).

## Conclusions

The study demonstrated that root morphological variation was the main adaptive strategy in maize in response to P deficiency under the present condition. A large root system is, therefore, important for maize to access available nutrients in soil, especially immobile P. Breeding crops such as maize with large root systems and better architecture will also contribute to increasing the use efficiency of P and other nutrients. NH_4_NO_3_ enhanced the magnitude of proton release in white lupin and faba bean under P deficiency although N form did not fundamentally alter the responses of root morphology and physiology of the three species to P deficiency. It is important in practices to use appropriate N forms to maximize root/rhizosphere processes and to enhance P-use efficiency. The use of ammonium-based fertilizers may facilitate P acquisition of legumes in non-acidic P-deficient soils but further work is needed to elucidate the effects of N form on P acquisition strategies of N_2_-fixing legumes.

## Sources of Funding

This research was supported by the State Key Basic Research and Development Plan of China (No. 2013CB127402).

## Contributions by the Authors

C.L. conceived and designed research; H.L. performed the experiments and analyzed data; C.L. coordinated the research, provided all the technical support, and participated in data interpretation; H.L., C.T. and C.L. wrote the article; all authors participated in stimulating discussion and approved the final article.

## Conflicts of Interest Statement

None declared.

## Supplementary Material

Supplementary Data
