# Supplementary material for: The effects of nitrogen form on root morphological and physiological adaptations of maize, white lupin and faba bean under phosphorus deficiency
Source: AoB Plants. 2016 Aug 12;8:plw058. doi: 10.1093/aobpla/plw058 (PMC5018397; doi:10.1093/aobpla/plw058)
Supplement: Supplementary Data [file supp_8_plw058_index.html]

The effects of nitrogen form on root morphological and physiological adaptations of maize, white lupin and faba bean under phosphorus deficiency — Supplementary Data 

# The effects of nitrogen form on root morphological and physiological adaptations of maize, white lupin and faba bean under phosphorus deficiency

## Supplementary Data

files

- Supplementary Data - docx file
- Supplementary Data - docx file
- Supplementary Data - doc file
