# Supplementary material for: The effects of nitrogen form on root morphological and physiological adaptations of maize, white lupin and faba bean under phosphorus deficiency
Source: AoB Plants. 2016 Aug 12;8:plw058. doi: 10.1093/aobpla/plw058 (PMC5018397; doi:10.1093/aobpla/plw058)
Supplement: Supplementary Data [file supp_plw058_aobplants-16011-s01.docx]

**Figure S1.**Dry weights of shoot and root of white lupin, faba bean and maize grown with low (LP, 1µmol L^-1^) and high P (HP, 250 µmol L^-1^) under two N forms for 7, 12 and 16 days. Nitrogen was supplied as Ca(NO_3_)_2_ on left panels and NH_4_NO_3_ on right panels. Values are means of four replicates, and the bars represent ± the standard error of the mean.

**Table S1. Nitrogen and potassium contents of intact white lupin, faba bean and maize grown with LP (1 µmol L^-1^) and HP (250 µmol L^-1^) under two N forms [Ca(NO_3_)_2_ and NH_4_NO_3_] for 7, 12 and 16 days.**

| N forms | P supply | White lupin | |  |  | Faba bean | |  |  | Maize |  |  |
| --- | --- | --- | --- | --- | --- | --- | --- | --- | --- | --- | --- | --- |
|  |  | 7d | 12d | 16d |  | 7d | 12d | 16d |  | 7d | 12d | 16d |
|  |  | N content (mg N per plant) | | | | | | | | | | |
| Ca(NO_3_)_2_-N | LP | 8.82 | 15.87 | 21.31 |  | 30.10 | 37.49 | 55.45 |  | 11.68 | 13.71 | 16.24 |
|  | HP | 9.37 | 14.31 | 22.20 |  | 25.00 | 40.49 | 65.89 |  | 10.42 | 30.32 | 57.39 |
| NH_4_NO_3_-N | LP | 9.51 | 12.23 | 25.50 |  | 31.57 | 49.02 | 64.74 |  | 13.75 | 17.60 | 23.51 |
|  | HP | 9.25 | 13.36 | 27.29 |  | 30.39 | 46.59 | 77.91 |  | 14.89 | 41.77 | 76.15 |
| LSD (*P* = 0.05) |  | 0.67 | 1.77 | 2.42 |  | 4.58 | 6.05 | 9.26 |  | 1.45 | 3.32 | 6.15 |
| P level |  | n.s. | n.s. | n.s. |  | n.s. | n.s. | ** |  | n.s. | *** | *** |
| N form |  | n.s. | * | ** |  | n.s. | ** | ** |  | *** | *** | *** |
| P × N |  | n.s. | n.s. | n.s. |  | n.s. | n.s. | n.s. |  | * | ** | ** |
|  |  | K content (mg K per plant) | | | | | | | | | | |
| Ca(NO_3_)_2_-N | LP | 5.09 | 10.86 | 24.32 |  | 21.21 | 33.30 | 55.25 |  | 22.74 | 27.59 | 44.29 |
|  | HP | 5.09 | 11.11 | 25.25 |  | 21.31 | 44.01 | 83.76 |  | 21.11 | 60.75 | 119.48 |
| NH_4_NO_3_-N | LP | 4.18 | 5.95 | 18.44 |  | 14.86 | 20.66 | 37.26 |  | 22.74 | 29.30 | 48.67 |
|  | HP | 4.12 | 5.40 | 18.23 |  | 16.50 | 26.41 | 59.56 |  | 23.74 | 61.22 | 146.44 |
| LSD (*P* = 0.05) |  | 0.42 | 0.92 | 2.54 |  | 2.66 | 4.52 | 9.23 |  | 2.66 | 5.12 | 10.52 |
| P level |  | n.s. | n.s. | n.s. |  | n.s. | *** | *** |  | n.s. | *** | *** |
| N form |  | *** | *** | *** |  | *** | *** | *** |  | n.s. | n.s. | ** |
| P × N |  | n.s. | n.s. | n.s. |  | n.s. | n.s. | n.s. |  | n.s. | n.s. | * |

**P* ≤0.05; ***P*<0.01; ****P*<0.001; n.s. not significant at *P*=0.05.
