# Supplementary material for: The effects of nitrogen form on root morphological and physiological adaptations of maize, white lupin and faba bean under phosphorus deficiency
Source: AoB Plants. 2016 Aug 12;8:plw058. doi: 10.1093/aobpla/plw058 (PMC5018397; doi:10.1093/aobpla/plw058)
Supplement: Supplementary Data [file supp_plw058_aobplants-16011-s02.docx]

1. **Topic select**

Nutrition

Rhizosphere (a suggested topic needs to be added to the list)

**2) Promotional statement**

Liu et al. compare the mechanisms of root adaptation of maize, white lupin and faba bean to phosphorus deficiency under supply of Ca(NO_3_)_2_ and NH_4_NO_3_. Phosphorus deficiency decreases shoot growth but increases root growth in maize and faba bean. It enhances the release of protons, organic acid anions and acid phosphatase from the roots of white lupin and faba bean. Compared with Ca(NO_3_)_2_, NH_4_NO_3_ dramatically increases proton release by roots but does not alter root morphology or physiology of the three species in response to phosphorus deficiency.

**3) Image**


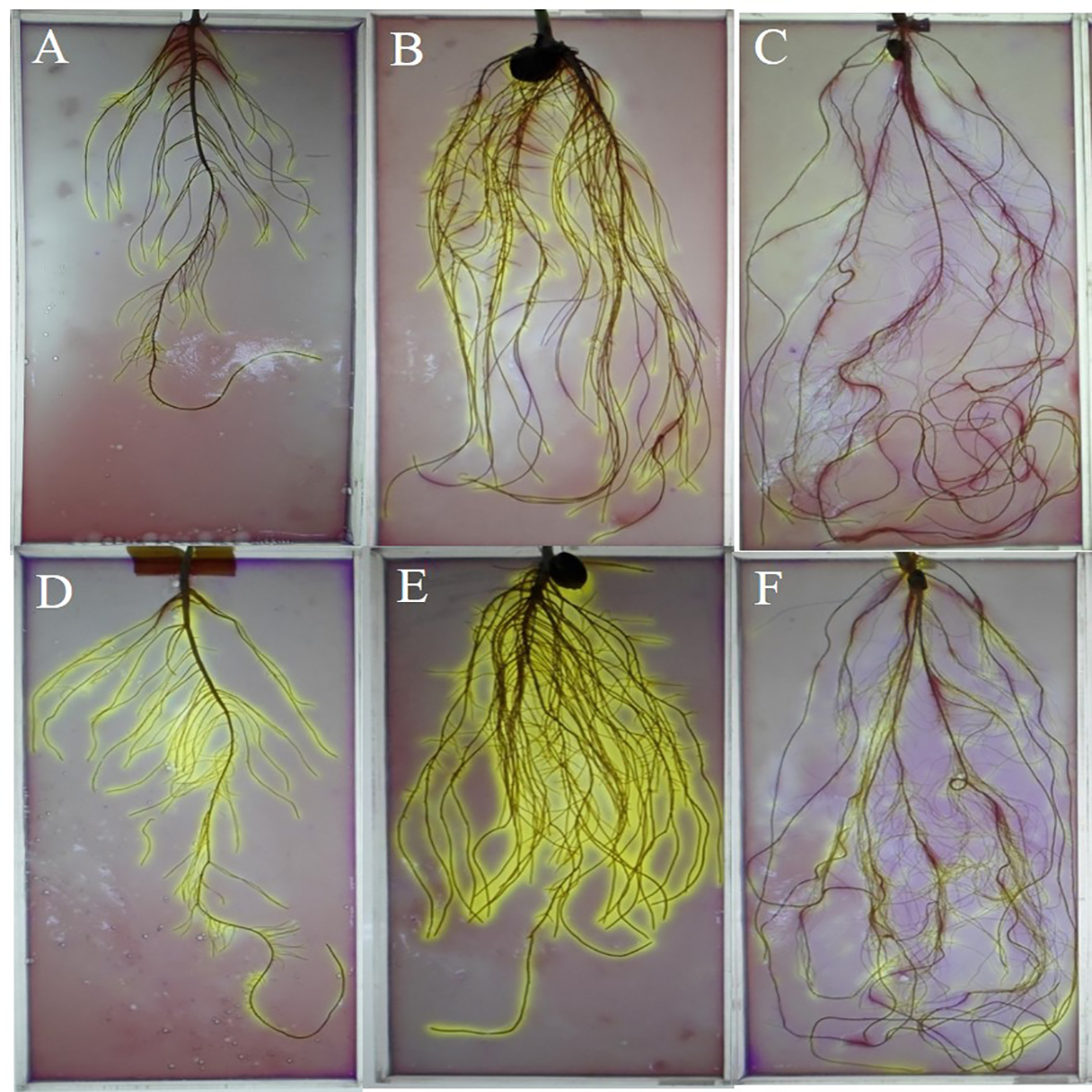


**Figure.** Effects of phosphorus deficiency (1 µmol L^-1^) on the intensity of rhizosphere acidification/alkalization in white lupin (A, D), faba bean (B, E) and maize (C, F) grown with Ca(NO_3_)_2_ (A, B, C) or NH_4_NO_3_ (D, E, F). Rhizosphere pH changes were detected by embedding the roots of 12-d-old plants in agar with bromocresol-purple as a pH indicator. The images were recorded 0.5 h after embedding. Yellow color indicates pH <5.2 while purple color indicates pH >6.8.

**4) A list of email addresses**

[Philip.White@hutton.ac.uk](mailto:Philip.White@hutton.ac.uk),

[fritschif@missouri.edu](mailto:fritschif@missouri.edu),

John.Kovar@ARS.USDA.GOV,

lindsey.227@osu.edu,

Yaping.Zhou@uni-hohenheim.de,

jbshen@cau.edu.cn,

hans.lambers@uwa.edu.au,

zhangyu9999666@163.com,

[philippe.hinsinger@supagro.inra.fr](mailto:philippe.hinsinger@supagro.inra.fr)

**5) The name of any Special Issue for which your paper is being prepared.**

No special issue.
