# Supplementary material for: The effects of nitrogen form on root morphological and physiological adaptations of maize, white lupin and faba bean under phosphorus deficiency
Source: AoB Plants. 2016 Aug 12;8:plw058. doi: 10.1093/aobpla/plw058 (PMC5018397; doi:10.1093/aobpla/plw058)
Supplement: Supplementary Data [file supp_plw058_aobplants-16011-s03.doc]

# OPEN ACCESS – RESEARCH ARTICLE

# The effects of nitrogen form on root morphological and physiological adaptations of maize, white lupin and faba bean under phosphorus deficiency

### Haitao Liu^1^, Caixian Tang ^2^, Chunjian Li^1^,*

^1^Department of Plant Nutrition, China Agricultural University, Beijing, 100193, China

^2^Department of Animal, Plant and Soil Sciences, La Trobe University, Bundoora (Melbourne) VIC 3086, Australia

*Corresponding author

Corresponding author’s e-mail address: [**lichj@cau.edu.cn**](mailto:lichj@cau.edu.cn)

Running head: Effects of N form on root responses of three plant species to P deficiency
